# TeleAllergy: Potential of Telemedicine in Management of Patients With Allergies

**DOI:** 10.2196/75483

**Published:** 2025-11-06

**Authors:** Hanna Lindemann, Emil Hammer, Luca Bonifacio, Christian Greis, Karin Hartmann

**Affiliations:** 1 Division of Allergy, Department of Dermatology, University Hospital Basel and University of Basel, Burgfelderstrasse 101, Basel, 4055, Switzerland, 41 612654080; 2 Department of Clinical Research, University Hospital Basel and University of Basel, Basel, Switzerland; 3 Department of Biomedicine, University Hospital Basel and University of Basel, Basel, Switzerland; 4 Department of Dermatology, University Hospital Zurich and University of Zurich, Zurich, Switzerland

**Keywords:** teleallergy, telemedicine, healthcare access, allergy management, teledermatology, synchronous communication, asynchronous communication, digital health, allergy diagnosis, teleconsultation, healthcare technology, patient-centered care

## Abstract

**Background:**

The growing prevalence of allergic diseases alongside a shortage of trained allergists creates significant challenges in delivering timely care, especially for underserved populations. Telemedicine presents a promising solution, offering remote care through digital tools. While telemedicine has been widely adopted in other fields, its use in allergy care remains underexplored.

**Objective:**

This study aimed to assess the potential of telemedicine in managing allergic diseases by examining patient preferences and experiences.

**Methods:**

A survey of 27 questions was distributed to adult patients (>18 y) with allergic diseases attending the outpatient allergy clinic at the Division of Allergy, University Hospital Basel, Basel, Switzerland, between May and August 2024. The survey covered demographic information, prior use of telemedicine, and preferences for teleconsultation modalities. It also assessed patients' willingness to share various types of clinical data, including images and written reports, and explored which allergic diseases were considered appropriate for telemedicine.

**Results:**

A total of 102 patients participated in the survey, with a mean age of 44.4 years (SD 16.7 y). For further analysis, the patients were stratified into four age groups: 18‐34 years (36/102), 35‐49 years (26/102), 50‐64 years (31/102), and ≥65 years (9/102). Among them, 44% (41/94; *P*=.22) had previously used telemedicine services, with 34% (32/94; *P*=.04) specifically using it for allergic diseases. When asked about consultation formats, 49% (49/100) of patients preferred in-person visits, while 41% (41/100) favored a hybrid model combining telemedicine and in-person care. Regarding telemedicine tools, 57% (51/89) preferred telephone consultations with a doctor. Patients would use telemedicine preferentially for mild compared to severe allergic diseases as well as for chronic compared to acute conditions. The spectrum of diseases for which patients would use telemedicine comprised a wide range of allergic conditions, with allergic rhinoconjunctivitis (16%; 14/85), Hymenoptera venom allergy (13%; 11/85), and food allergy/intolerance (13%; 11/85) cited most frequently. Only 7% (6/85) of patients indicated they would not use telemedicine for any allergic disease.

**Conclusions:**

This study emphasizes the growing adoption and importance of telemedicine in allergy care, with a significant proportion of patients already having experience using it for managing allergic diseases. Patients’ inclination toward multiple communication formats underscores the growing need for individualized management of allergic diseases.

## Introduction

### Challenges in Allergy Care

The increasing prevalence of allergic diseases, combined with a persistent shortage of trained allergists, presents significant challenges in delivering timely and accessible allergy care. This issue is especially pronounced in underserved rural areas and among vulnerable populations, such as older adults and those with mobility impairments [1,2].

Allergic diseases encompass a wide spectrum of conditions, from common diseases such as allergic rhinitis, eczema, and food allergy, to rare and frequently severe disorders such as mastocytosis and hereditary angioedema. Despite their varying severity, these diseases share the need for specialized expertise and ongoing, long-term management [1,2].

### Potential of Telemedicine

Telemedicine, particularly during the COVID-19 pandemic, has emerged as a promising and cost-effective solution to address these challenges, utilizing digital technologies to connect patients with health care providers. It offers two primary modes of communication: synchronous, where patients and providers interact in real-time (such as video consultations) and asynchronous, which allows communication at different times. A widely used asynchronous method is store-and-forward technology, where clinical data, such as images or medical records, are uploaded in a digital patient folder for later review. This approach has been particularly effective in teledermatology, where visual assessments are crucial [3-5].

In addition to communication tools, various digital applications and technologies are available to support health care management, such as e-diaries (“digital companion”) that track symptoms and therapy adherence, as well as electronic prescriptions and appointment scheduling systems that streamline health care processes [6].

While telemedicine is well-established in many medical specialties, there is limited data on its application for patients with allergies. In this study, we employed a patient-centered questionnaire to evaluate the current use of telemedicine in allergy care, to identify patients’ preferred communication tools and the specific allergic conditions for which they would be open to using telemedicine.

### Objective

This study aimed to assess telemedicine’s role in allergy care by evaluating patient preferences, usage patterns, and conditions considered suitable for remote management.

## Methods

### Study Design and Participants

To evaluate patient experiences and preferences with telemedicine, we conducted a survey of 27 questions targeting adult patients (over 18 y) with various allergic diseases prior to their consultations at our outpatient allergy clinic. Exclusion criteria included language barriers, cognitive impairment, and lack of informed consent.

### Inclusion and Exclusion Criteria

The questionnaire, developed in German, French, Italian, and English, collected demographic data, educational background, travel time to the nearest allergist, prior use of telemedicine services, the extent of health insurance coverage for telemedicine, and patients’ preferences regarding teleconsultation modalities. It also explored which digital communication tools and clinical data types (eg, images or medical reports) patients were open to sharing. Furthermore, the survey assessed which allergic diseases (categorized as mild or severe and acute or chronic based on patients’ subjective evaluation of their symptoms) patients considered appropriate for telemedicine. Questions were formatted as binary responses, multiple-choice items, visual analog scales (0‐10), and open-ended text fields to capture comprehensive insights.

### Statistical Analysis

To analyze the data collected through the questionnaire across different demographic categories, the 102 patients were categorized into four age groups: 18‐34 years, 35‐49 years, 50‐64 years, and over 65 years. For continuous variables such as age, the mean and standard deviation were calculated, while categorical variables are presented as absolute frequencies (n) and percentages (%). Group differences in telemedicine usage and preferences were evaluated using *χ*^2^ tests, with Bonferroni correction applied to adjust the family-wise error rate in multiple pairwise comparisons. Statistical significance was defined as a two-sided *P* value <.05. All analyses were conducted using R software (version 4.2.0; R Foundation for Statistical Computing).

### Ethical Considerations

The study was reviewed by the Ethics Committee of Northwest and Central Switzerland (EKNZ). According to their assessment (Ref: Req-2024‐00650), the project does not fall under the Swiss Human Research Act, as only anonymized data were processed, and therefore no formal approval was required.

## Results

### Demographics

Between May 29, 2024, and August 8, 2024, a total of 102 patients completed the questionnaire (mean age 44.4, SD 16.7 y). Age distribution was as follows: 35% participants (36/102) were 18‐34 years old, 25% (26/102) were 35‐49 years, 30% (31/102) were 50‐64 years, and 9% (9/102) were ≥65 years. 61% were women (62/102). Most of the respondents lived in suburban areas with populations between 10,000 and 1,00,000 (37/101; 36%) or in rural areas with fewer than 10,000 inhabitants (36/101; 35%). Approximately, 57% participants (58/101) reported a travel time of 10 to 29 minutes to reach an allergist, and 49% (50/102) held a university or college degree (Table S1 in Multimedia Appendix 1).

### Experience With Telemedicine

**Figure 1.**
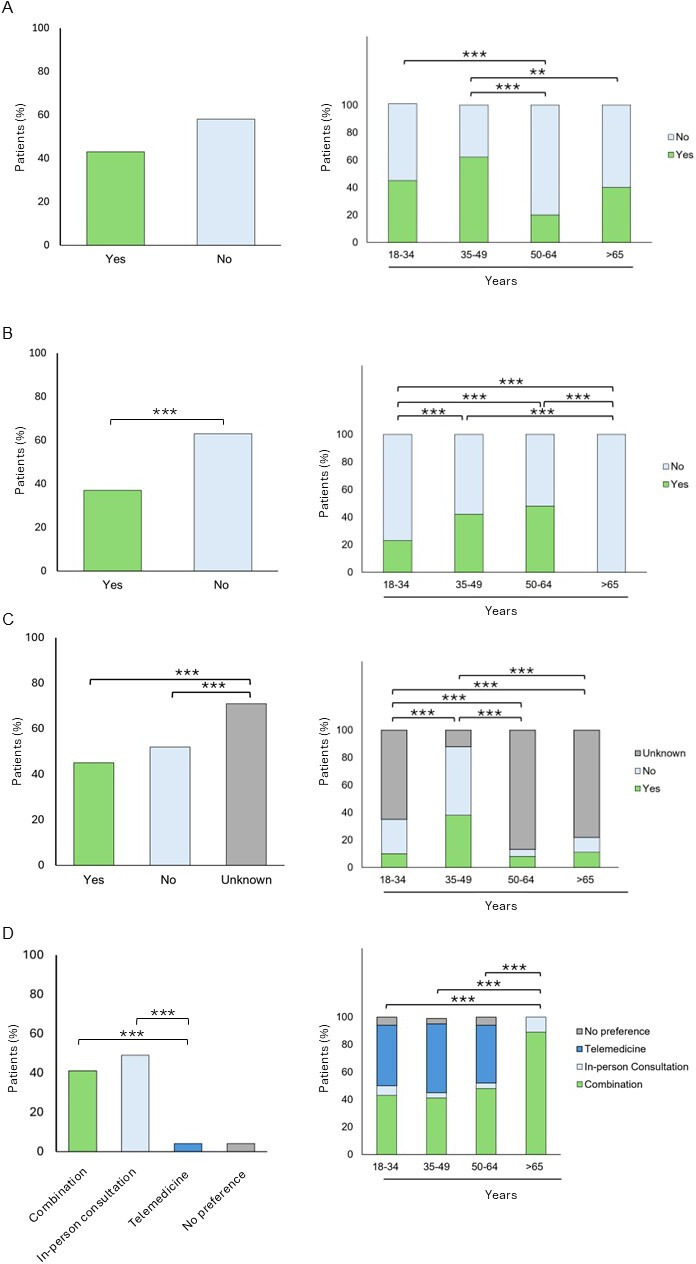
Patients’ experience and preferences in telemedicine. (A) Patients (%) with previous experience in telemedicine. (B) Patients (%) with previous experience in telemedicine consultations for allergic conditions. (C) Patients (%) with insurance coverage for telemedicine. (D) Patients (%) by preferred consultation method. Levels of statistical significance are indicated as follows: * *P*< .05; ** *P*< .01; *** *P*< .001.

44% of the respondents (41/94; *P*=.22) reported previous use of telemedicine services. Prior telemedicine use was significantly associated with age group (*P*<.001), as was prior use of tele-allergy services (*P*<.001). The highest rate of telemedicine use was observed in the 35‐49 age group (16/26; 62%), significantly higher than in the 50‐64 group (*P*<.001) and the ≥65 group (*P*=.04) (Figure 1A). 39% patients (37/94; *P*=.04) had used telemedicine specifically for allergy-related care (Figure 1B). Prior tele-allergy use was significantly associated with age group (*P*<.001), and was most frequent in the 50‐64 age group (14/30; 47%), significantly more than in the 18‐34 group and the ≥65 group (both *P*<.001).

### Awareness of Insurance Coverage

Uncertainty regarding insurance coverage for telemedicine was high, with 71% (73/102) unsure about reimbursement. This proportion was significantly greater than among those who responded with “yes” or “no” (*P*<.001). Awareness differed by age group (*P*<.001), with only 12% (3/26) of the 35‐49 age group reporting uncertainty compared to all other age groups (*P*<.001) (Figure 1C).

### Preferences for Consultation Formats

49% respondents (49/100) preferred exclusively in-person consultations (*P*=.04), whereas 41% (41/100) favored a hybrid model (*P*=.07) compared to telemedicine. Age-related differences in preference were significant (*P*<.001), with the ≥65 group being significantly less inclined to use telemedicine than all younger groups (*P*<.001) (Figure 1D).

### Preferred Telemedicine Communication Tools and Consultation Formats

A significantly greater number of patients (70/92; 76%; *P*<.001) expressed interest in sharing photos and images with health care providers, whereas 61% patients (56/92; *P*=.01) preferred sharing brief written reports (<5 pages) (Table S2 in Multimedia Appendix 1).

A statistically significant association was observed between age and the preference for sharing photos or brief written reports (*P*<.001). All younger age groups showed a significantly higher preference for these communication methods (*P*<.001) compared to participants aged ≥ 65 years (data not shown).

The most favored telemedicine communication formats included phone calls with doctors (51/89; 57%; *P*=.02). Asynchronous modes such as email/chat with trained staff (27/89; 30%; *P*=.002) or physicians (24/89; 27%; *P*=.0014), video consultations with trained staff (33/89; 37%, *P*=.03), and digital patient information like instruction videos (27/89; 30%; *P*=.002) and symptom diaries (24/89; 27%; *P*=.0014) were significantly less preferred. Of these, preferences for phone calls with doctors differed significantly by age (*P*<.001). Only 4% (2/89) of participants aged ≥65 endorsed phone calls this consultation format, compared to those aged 18‐34 (*P*<.001) (data not shown).

### Allergic Conditions in Telemedicine

Telemedicine was considered especially helpful for managing mild allergic conditions (70/100; 70%; *P*=.007) and chronic conditions (48/100; 48%; *P*=.84), while considerably fewer participants supported its use for severe (11/100; 11%; *P*<.001) or acute conditions (27/100; 27%; *P*<.001).

**Figure 2.**
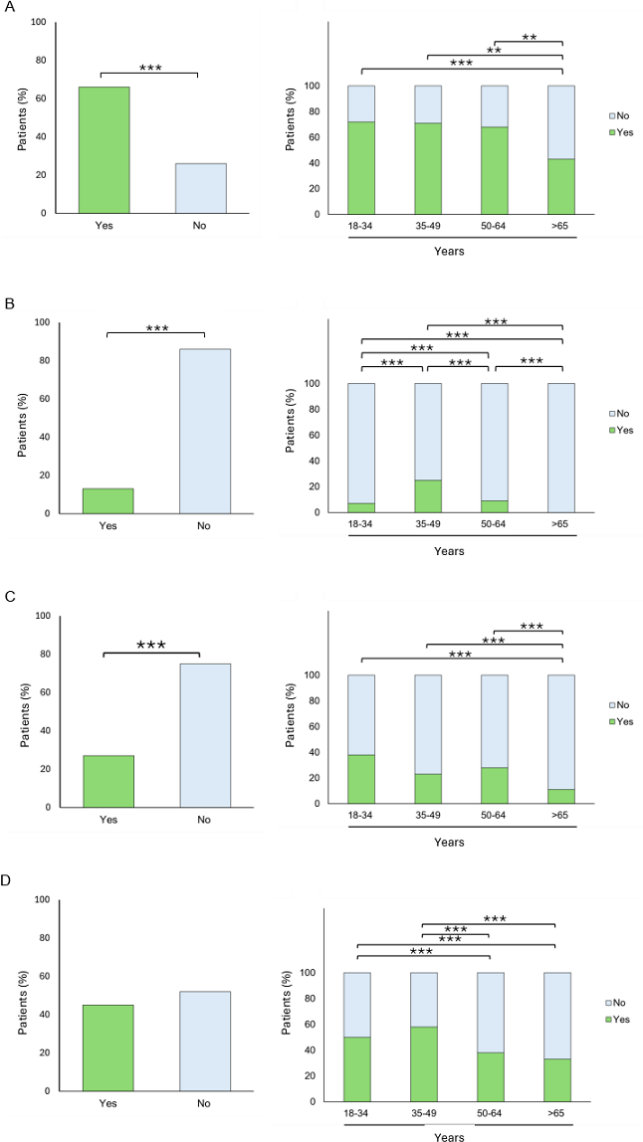
Patients’ willingness to use telemedicine based on the type of allergic disease. (A) Patients (%) willing to use telemedicine for mild allergic diseases. (B) Patients (%) willing to use telemedicine for severe allergic diseases. (C) Patients (%) willing to use telemedicine for acute allergic diseases. (D) Patients (%) willing to use telemedicine for chronic allergic diseases. Levels of statistical significance are indicated as follows: *P< .05; **P< .01; ***P< .001.

Participants aged ≥65 were significantly less likely to consider telemedicine appropriate for mild allergic conditions compared to all younger age groups (Figure 2A).

For severe allergies, individuals aged 35‐49 reported a significantly higher willingness to use telemedicine compared to all other age groups (all *P*<.001) (Figure 2B).

For acute conditions, adults aged ≥65 again reported a significantly lower willingness to use telemedicine compared to all other age groups (all *P*<.001) (Figure 2C).

Regarding chronic diseases, patients aged between 18‐34 years, reported a significantly higher willingness to use telemedicine compared to individuals aged 50‐64 and ≥65 (both *P*<.001) (Figure 2D).

The spectrum of allergic diseases for which patients would use telemedicine encompassed allergic rhinoconjunctivitis (16.47%; 14/85), Hymenoptera venom allergy (12.94%; 11/85), and food allergy/intolerances (12.94%; 11/85) as most frequent diseases, followed by various less frequent allergic conditions (Multimedia Appendix 2).

Only a minority of patients (7.06%; 6/85) indicated they would not use telemedicine for any allergic disease (data not shown).

## Discussion

### Principal Findings

Our study demonstrates the increasing role of telemedicine in allergy care. We found high adoption rates of telemedicine, especially among younger patients, and a preference for hybrid models that combine telemedicine with in-person consultations. Telemedicine in allergy care is seen by patients as especially useful for managing mild conditions.

### Demographics

Demographic data on age and gender in allergy patient surveys are limited, but our findings align with previous telemedicine studies, showing a mean age of 44.4 years and a higher participation rate among women [3]. The proportion of urban residents in our cohort was slightly lower compared to data from the Swiss Federal Statistical Office [7], likely due to the lower density of allergists in rural areas, requiring travel to urban centers for consultations [8].

### Experience With Telemedicine

A high percentage of respondents reported using telemedicine services (Figure 1), consistent with findings from other medical fields [3,9]. This increased adoption of telemedicine in our cohort may partly be explained by the higher educational attainment often associated with higher usage rates [9]. Patients aged 35‐49 years were the most frequent users of telemedicine (Figure 1), aligning with previous research. Interestingly, those aged 65 and older used telemedicine services more than those aged 50‐65 years, suggesting an unmet need for digital health services among older, potentially less mobile patients—a trend also observed in other studies [10]. However, given the small sample of patients aged 65 and older (9 patients), this finding should be interpreted cautiously.

Although we could not determine the specific share of allergy services within telemedicine offerings, allergy services were not typically emphasized in studies assessing usage rates across specialties [11]. The high utilization rates in our study may also be influenced by selection bias, as participants were surveyed in the waiting area of the outpatient allergy clinic.

### Awareness of Insurance Coverage

Despite 29 of the 37 Swiss health insurance providers offering telemedicine policies and basic insurance (OKP) covering telemedicine services when proven effective, 71% (73/102) of patients in our cohort were unaware of their coverage [12]. This suggests an unmet need for allergy services, as many patients may be paying out-of-pocket for telemedicine. On the other hand, younger patients, who often opt for insurance plans with higher deductibles, may find telemedicine more cost-effective than in-person visits. This trend is likely influenced by the Swiss health care system, which allows for flexible deductible options.

Older patients tend to prefer in-person consultations, likely due to concerns about remote care quality, limited familiarity with technology, and reliance on face-to-face interactions for building trust [13,14]. In contrast, 41% (41/100) of all patients preferred a hybrid model that combines telemedicine with in-person visits, while only 8% (8/100) favored telemedicine exclusively. This suggests that digital services are seen as complementary to, rather than a replacement for, traditional in-person care, enhancing access and alleviating pressure on health care systems [4,9]. Younger patients (18‐34 y) preferred telephone consultations, particularly when integrated with video or in-person visits for comprehensive assessment. In contrast, patients over 50 years favored video consultations by a doctor (44%), likely due to their preference for face-to-face interactions [5,15].

Digital health tools, including online appointment scheduling and e-prescriptions, were widely embraced by patients aged 18‐49, indicating higher comfort with technology in these age groups. However, adoption was lower among patients over 65, likely due to barriers such as unfamiliarity with digital platforms and concerns about data security [15-17].

The field of allergy care lacks substantial evidence on the most useful communication types for telemedicine consultations [5]. In our study, most patients expressed interest in uploading photos, a preference similar to findings from dermatology, where visual information is crucial for remote consultations [4]. Over half of the patients in our study preferred sharing detailed written reports, reflecting the complex and heterogeneous nature of allergic diseases, which may require more individualized data exchanges.

### Allergic Conditions in Telemedicine

Patients in our survey expressed a preference for telemedicine in managing mild and chronic allergic diseases (Figure 2), aligning with broader trends in telemedicine across specialties [18]. While a small proportion of patients expressed concerns about using telemedicine for severe allergic diseases, some studies suggest that telemedicine can positively impact outcomes for diseases like anaphylaxis by providing specific management instructions [19]. However, telemedicine should not be considered a substitute for emergency care. It can play an important role in managing allergies, but cannot replace emergency responses or in-person care. Given that many allergic diseases are chronic and benefit from ongoing monitoring, telemedicine represents an effective approach for long-term management, a view that was supported by most participants in our study.

### Limitations

While our study offers valuable preliminary insights into telemedicine use in allergy care, several limitations should be considered when interpreting the findings.

First, the sample size of 102 participants, though sufficient for initial exploration, limits the statistical power, particularly for subgroup analyses by diagnosis and age groups. The small number of respondents aged 65 and older further restricts conclusions for this demographic, which is particularly relevant given their lower engagement with telemedicine.

Second, recruitment was conducted at a single center and participation was voluntary, which may have introduced self-selection bias. Individuals with a greater interest or familiarity with digital health tools may have been more likely to participate, potentially skewing the findings and limiting generalizability to broader patient populations.

Third, the study population included both new and follow-up patients. While this reflects real-world clinical diversity, differences in experience and expectations between these groups may have influenced their responses, particularly regarding telemedicine preferences and awareness of insurance coverage. To address these limitations, future studies should include larger and more diverse samples across multiple sites.

### Conclusion

In conclusion, our study highlights the growing role of telemedicine in allergy care. High adoption rates, especially among younger patients, suggest that telemedicine can complement in-person care. Future retrospective research is needed to refine teleconsultation strategies, enhancing diagnostic accuracy, therapeutic effectiveness, and patient satisfaction, thereby maximizing patients’ benefits from telemedicine in allergy care.

## Supplementary material

10.2196/75483 Multimedia Appendix 1 Distribution of patients by age group, gender, residence, distance to allergist, and education level and patients' preferences for digital communication and telemedicine consultations for allergic diseases.

10.2196/75483 Multimedia Appendix 2 Allergic diseases for which patients would use telemedicine.
