# Supplementary material for: TeleAllergy: Potential of Telemedicine in Management of Patients With Allergies
Source: JMIR Hum Factors. 2025 Nov 6;12:e75483. doi: 10.2196/75483 (PMC12591358; doi:10.2196/75483)
Supplement: Multimedia Appendix 1 [file humanfactors-v12-e75483-s001.docx]

**Table 1:** Patients (n=102) by age group, gender, residence, distance to allergist, and education level.

| Variable | | Patients, n (%) | Age group by years, n (%) | | | |
| --- | --- | --- | --- | --- | --- | --- |
|  |  |  | 18–34 | 35–49 | 50–64 | ≥ 65 |
| **Gender, (n = 102)** | | | | | | |
|  | Female | 62  (60.8) | 22 (35.5) | 15 (24.2) | 19 (30.6) | 6  (9.7) |
|  | Male | 40  (39.2) | 14 (35.0) | 11 (27.5) | 12 (30.0) | 3  (7.5) |
|  | Diverse | 0  (0.0) | – | – | – | – |
|  | Not available | 0  (0.0) | – | – | – | – |
| **Place of residence, (n = 101)** | | | | | | |
|  | Urban (> 100,000) | 28  (27.7) | 10 (35.7) | 8 (28.6) | 8 (28.6) | 2  (7.4) |
|  | Sub-Urban (10,000–100,000) | 37  (36.6) | 16 (43.2) | 7 (18.9) | 10 (27.0) | 4 (10.8) |
|  | Rural (< 10,000) | 36  (35.6) | 9  (25.0) | 11 (30.6) | 13 (36.1) | 3  (8.3) |
|  | Not available | 1  (1.0) | 1 (100.0) | – | – | – |
| **Distance to allergist, (n = 101)** | | | | | | |
|  | < 10 min | 14  (13.9) | 5  (35.7) | 3 (21.4) | 5 (35.7) | 1  (7.1) |
|  | 10–29 min | 58  (57.4) | 22 (37.9) | 14 (24.1) | 15 (25.9) | 7 (12.1) |
|  | 30–59 min | 21  (20.8) | 6  (28.6) | 7  (33.3) | 7 (33.3) | 1  (4.8) |
|  | 60–90 min | 6  (5.9) | 2  (33.3) | 1  (16.7) | 3 (50.0) | 0  (0.0) |
|  | > 90 min | 2  (2.0) | 0  (0.0) | 1  (50.0) | 1 (50.0) | 0  (0.0) |
|  | Not available | 1  (1.0) | 1 (100.0) | – | – | – |
| **Education level, (n = 102)** | | | | | | |
|  | Primary/secondary school | 6  (5.9) | 2  (33.3) | 2 (33.3) | 1 (16.7) | 1 (16.7) |
|  | High school diploma | 14  (13.7) | 9  (64.3) | 4 (28.6) | – | 1  (7.1) |
|  | Vocational training | 32  (31.4) | 8  (25.0) | 9 (28.1) | 12 (37.5) | 3  (9.4) |
|  | University/college degree | 50  (49.0) | 17 (34.0) | 11 (22.0) | 18 (36.0) | 4  (8.0) |
|  | Not available | - | – | – | – | – |

**Table 2:** Patients' (n=102) preferences for digital communication and telemedicine consultations for allergic diseases.

| Variable | | Patients, n (%) | Age group by years, n (%) | | | |
| --- | --- | --- | --- | --- | --- | --- |
|  |  |  | 18–34 | 35–49 | 50–64 | 18–34 |
| **Preferred telemedicine communication tools, (n = 92)** | | | | | | |
|  | Written report < 5 pages | 56  (60.9)  p = .011 | 23  (41.1) | 16  (28.6) | 13 (23.2) | 4  (7.4) |
|  | Written report 5–10 pages | 26  (28.3)  p = 1.00 | 10  (38.5) | 8  (30.8) | 6  (23.1) | 2  (7.7) |
|  | Written report > 10 pages | 26  (28.3)  p = 1.00 | 10  (38.5) | 5  (19.2) | 9  (34.6) | 2  (7.7) |
|  | Photos/images | 70  (76.1)  p < .001 | 27  (38.6) | 15  (21.4) | 23 (32.9) | 5  (7.1) |
|  | Short video (~15 seconds) | 28  (28.3)  p = .093 | 12  (42.9) | 8  (28.6) | 7  (25.0) | 1  (3.6) |
|  | Voice message | 28  (28.3)  p = .093 | 8  (28.6) | 6  (21.4) | 13 (46.4) | 1  (3.6) |
|  | Digital patient diary | 23  (25.0)  p = .87 | 7  (30.4) | 2  (8.7) | 13  (56.5) | 1  (4.3) |
|  | Not available | 10  (10.9)  p = .19 | 1  (10.0) | 5  (50.0) | 1  (10.0) | 3  (30.0) |
| **Preferred telemedicine consultation preferences, (n = 89)** | | | | | | |
|  | Telephone by trained staff | 48  (53.9)  *p = .46* | 21 (43.8) | 10 (20.8) | 16 (33.3) | 1  (2.1) |
|  | Video by trained staff | 33  (37.1)  p = .031 | 13 (39.4) | 7  (21.2) | 11 (33.3) | 2  (6.1) |
|  | Email/chat by trained staff | 27  (30.3)  p = .002 | 9  (33.3) | 5  (18.5) | 10 (37.0) | 3  (11.1) |
|  | Telephone by doctor | 51  (57.3)  p = .017 | 23 (45.1) | 14 (27.5) | 12 (23.5) | 2  (3.9) |
|  | Video by doctor | 47  (52.8)  p = .052 | 15 (31.9) | 11 (23.4) | 16 (34.0) | 5  (10.6) |
|  | Email/chat by doctor | 24  (27.0)  p = .0014 | 10  (41.7) | 6  (25.0) | 6 (25.0) | 2  (8.3) |
|  | Digital appointment booking for in-person consultation | 43  (48.3)  p = .076 | 21  (48.8) | 13 (30.2) | 9  (20.9) | – |
|  | E‑prescription | 42  (47.2)  p =0.57 | 24 (57.1) | 12 (28.6) | 6  (14.3) | – |
|  | Digital patient information (e.g., instructional videos) | 27  (30.3)  p ≤ .002 | 14 (51.9) | 5  (18.5) | 8  (29.6) | – |
|  | Digital patient diary for therapy/symptom assessment | 24  (27.0)  p ≤ .002 | 12 (50.0) | 8  (33.3) | 4  (16.7) | – |
|  | Not available | 13  (14.6)  *p < .0001* | 2  (15.4) | 5  (38.5) | 5  (38.5) | 1  (7.7) |
