# Supplementary figures and images for: TeleAllergy: Potential of Telemedicine in Management of Patients With Allergies
Source: JMIR Hum Factors. 2025 Nov 6;12:e75483. doi: 10.2196/75483 (PMC12591358; doi:10.2196/75483)

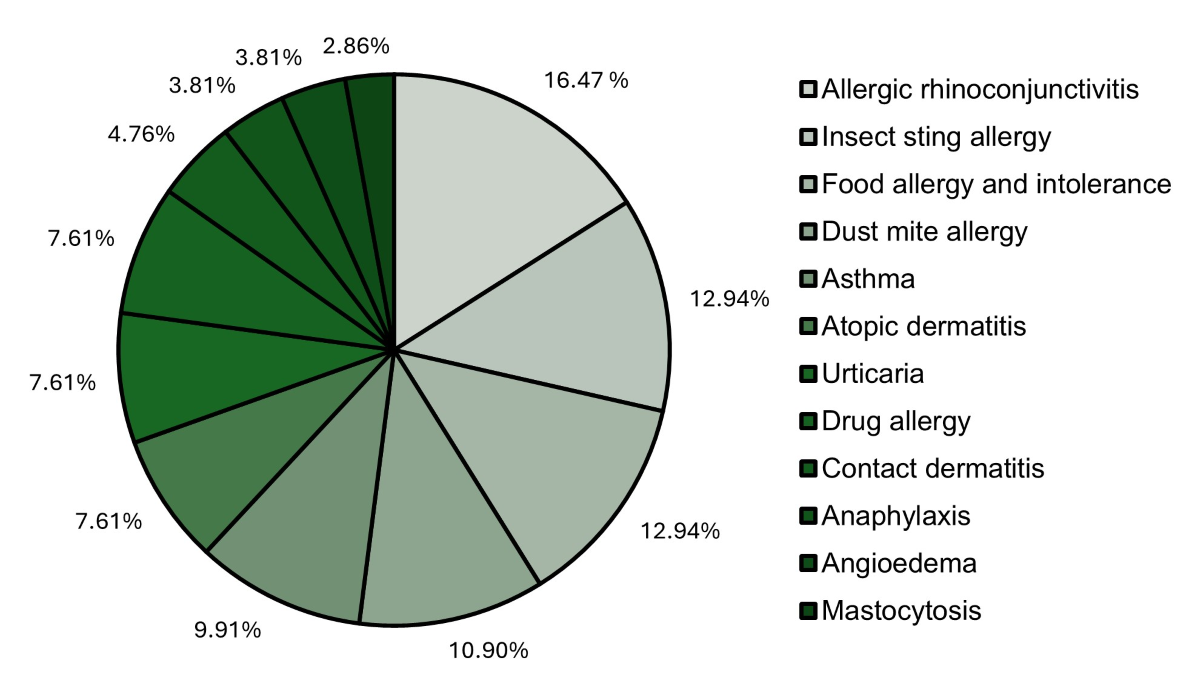

Supplement: Multimedia Appendix 2 [file humanfactors-v12-e75483-s002.png]
